# Cerebellar Volumes and Sensorimotor Behavior in Autism Spectrum Disorder

**DOI:** 10.3389/fnint.2022.821109

**Published:** 2022-05-03

**Authors:** Walker S. McKinney, Shannon E. Kelly, Kathryn E. Unruh, Robin L. Shafer, John A. Sweeney, Martin Styner, Matthew W. Mosconi

**Affiliations:** ^1^Schiefelbusch Institute for Life Span Studies and Kansas Center for Autism Research and Training (K-CART), University of Kansas, Lawrence, KS, United States; ^2^Clinical Child Psychology Program, University of Kansas, Lawrence, KS, United States; ^3^Department of Psychology, University of Kansas, Lawrence, KS, United States; ^4^Department of Psychiatry and Behavioral Neuroscience, University of Cincinnati College of Medicine, Cincinnati, OH, United States; ^5^Department of Psychiatry and Computer Science, University of North Carolina at Chapel Hill, Chapel Hill, NC, United States

**Keywords:** cerebellum, volumetry, autism spectrum disorder (ASD), sensorimotor, oculomotor, MRI, structure, Crus I

## Abstract

**Background:**

Sensorimotor issues are common in autism spectrum disorder (ASD), though their neural bases are not well understood. The cerebellum is vital to sensorimotor control and reduced cerebellar volumes in ASD have been documented. Our study examined the extent to which cerebellar volumes are associated with multiple sensorimotor behaviors in ASD.

**Materials and Methods:**

Fifty-eight participants with ASD and 34 typically developing (TD) controls (8–30 years) completed a structural MRI scan and precision grip testing, oculomotor testing, or both. Force variability during precision gripping as well as absolute error and trial-to-trial error variability of visually guided saccades were examined. Volumes of cerebellar lobules, vermis, and white matter were quantified. The relationships between each cerebellar region of interest (ROI) and force variability, saccade error, and saccade error variability were examined.

**Results:**

Relative to TD controls, individuals with ASD showed increased force variability. Individuals with ASD showed a reduced volume of cerebellar vermis VI-VII relative to TD controls. Relative to TD females, females with ASD showed a reduced volume of bilateral cerebellar Crus II/lobule VIIB. Increased volume of Crus I was associated with increased force variability. Increased volume of vermal lobules VI-VII was associated with reduced saccade error for TD controls but not individuals with ASD. Increased right lobule VIII and cerebellar white matter volumes as well as reduced right lobule VI and right lobule X volumes were associated with greater ASD symptom severity. Reduced volumes of right Crus II/lobule VIIB were associated with greater ASD symptom severity in only males, while reduced volumes of right Crus I were associated with more severe restricted and repetitive behaviors only in females.

**Conclusion:**

Our finding that increased force variability in ASD is associated with greater cerebellar Crus I volumes indicates that disruption of sensory feedback processing supported by Crus I may contribute to skeletomotor differences in ASD. Results showing that volumes of vermal lobules VI-VII are associated with saccade precision in TD but not ASD implicates atypical organization of the brain systems supporting oculomotor control in ASD. Associations between volumes of cerebellar subregions and ASD symptom severity suggest cerebellar pathological processes may contribute to multiple developmental challenges in ASD.

## Introduction

Autism spectrum disorder (ASD) is a neurodevelopmental disability for which brain mechanisms are not well understood. Sensorimotor difficulties are present in 70–80% of individuals with ASD (Dewey et al., 2007; Green et al., 2009) and are predictive of functional outcomes, including daily living skills (Travers et al., 2017). They also represent promising targets for advancing understanding of the underlying brain mechanisms of ASD because they are: (1) supported by cortical-cerebellar networks that are well-defined through animal and human lesion studies (Smith et al., 1981; Molinari et al., 1997; Hilber et al., 1998; Brochier et al., 1999; Kelly and Strick, 2003) and consistently implicated in ASD [for reviews, see Fatemi et al. (2012) and Mosconi et al. (2015b)]; (2) familial, suggesting that they may serve as endophenotypes representing polygenic risk (Mosconi et al., 2010; Mous et al., 2017), and; (3) associated with core features of ASD (Murray et al., 2006; LeBarton and Iverson, 2016; Travers et al., 2017; Iverson et al., 2019). Clarifying neuroanatomical substrates associated with sensorimotor differences in ASD is therefore important for understanding the pathophysiological mechanisms associated with the disorder(s).

Individuals with ASD show sensorimotor differences across effector systems, including skeletomotor and oculomotor systems. Multiple studies have documented reduced control of skeletomotor behavior in ASD including increased variability of both upper (Mosconi et al., 2015a; Wang et al., 2015; Unruh et al., 2021) and lower limb behavior (Glazebrook et al., 2006; Marko et al., 2015). Atypical oculomotor function in ASD has also been documented, including reduced accuracy and increased amplitude variability of saccades (Takarae et al., 2004; Stanley-Cary et al., 2011; Schmitt et al., 2014). These findings converge to suggest that individuals with ASD demonstrate alterations in sensorimotor processes spanning multiple effector systems and multiple types of behavior, including both sustained actions and rapid, ballistic movements.

Both sustained and rapid sensorimotor behaviors are supported by well-defined cortical, subcortical, and cerebellar systems. Cerebellum is particularly important for refining motor output through the comparison of internal predictive models of initial motor plans (i.e., “feedforward models”) and sensory feedback error information (Bastian, 2006; Shadmehr and Krakauer, 2008). During skeletomotor control, the lateral cerebellum (Crus I) integrates sensory feedback with feedforward models to refine motor output *via* anterior cerebellum (lobules I-V), lobule VI, and afferent relays to the primary motor cortex (M1) *via* the thalamus (Stein, 1986; Stein and Glickstein, 1992; Glickstein, 2000; Vaillancourt et al., 2006). Crus I plays a role in multiple motor and non-motor functions (Stoodley and Schmahmann, 2009; Stoodley et al., 2012), suggesting that it supports internal model representations and refinements across multiple neural systems (Kelly and Strick, 2003; Ito, 2008). Within the motor domain, reciprocal connections between cerebellar Crus I and M1 form a closed-loop circuit that supports the online refinement of endpoint target selection during movement (Proville et al., 2014). Reciprocal connections between M1 and cerebellar lobule VIII, which houses a secondary somatotopic representation, also support the refinement of motor output, largely as a supplement to anterior cerebellum and Crus I during the early stages of sensorimotor learning due to enhanced task demands (Steele and Penhune, 2010; Kuper et al., 2014; Bonzano et al., 2015).

Discrete oculomotor cerebellar networks support accurate eye movements generated in response to visual stimuli. Visually guided saccadic and smooth pursuit eye movements are generated through projections from the posterior vermal lobules VI-VII to caudal fastigial nuclei and subsequent execution by abducens motoneurons innervating the lateral rectus muscles (Ohtsuka and Noda, 1992; Scudder et al., 2002). Across skeletomotor and oculomotor cerebellar networks, white matter tracts support the integration of motor and sensory information *via* intracerebellar, afferent (through middle cerebellar peduncles), and efferent (through superior cerebellar peduncles) pathways (Salmi et al., 2010; Roberts et al., 2013; Koppelmans et al., 2015).

Structural MRI studies in ASD implicate multiple cerebellar subregions important for skeletomotor and oculomotor control. Within cerebellar Crus I, both increased (Stoodley, 2014; D’Mello et al., 2016) and decreased volumes have been reported (Yu et al., 2011; Duerden et al., 2012), though separate studies have not shown differences between individuals with ASD and TD controls (Nickl-Jockschat et al., 2012; DeRamus and Kana, 2015). Cerebellar lobules I-V show decreased volumes in individuals with ASD relative to typically developing (TD) controls (Allen et al., 2004; Duerden et al., 2012; Marko et al., 2015), and hemispheric lobule VI shows increased volumes in ASD relative to TD controls (Nickl-Jockschat et al., 2012). Structural differences in vermal lobules associated with oculomotor control also have been documented in ASD, including reduced volumes of vermal lobules VI-VII (Courchesne et al., 2001; Kaufmann et al., 2003; Stanfield et al., 2008; Webb et al., 2009; Crucitti et al., 2020), though others have suggested that vermal hypoplasia may be specific to individuals with (Stanfield et al., 2008) or without (Scott et al., 2009) comorbid intellectual/developmental disability. These structural MRI findings not only implicate dysmorphology of cerebellar lobules important for skeletomotor and oculomotor control in ASD, but also suggest patterns of cerebellar structural variation differ across separate lobules and as a function of clinical or behavioral characteristics (e.g., intellectual/developmental disability).

While several studies have examined the associations between cerebral regional volumes and sensorimotor abilities in ASD (Mostofsky et al., 2007; Mahajan et al., 2016; Lin et al., 2019), only one known study has assessed the covariation of cerebellar morphometry and skeletomotor behavior in ASD, and no known studies have examined the relationships between volumetrics of different cerebellar subregions and multiple separate sensorimotor behaviors in ASD (e.g., skeletomotor and oculomotor). During a reaching test, Marko et al. (2015) demonstrated that children with ASD show a reduced ability to adapt to visual perturbations and that these difficulties are associated with reduced volumes of bilateral cerebellar lobules I-V, VI, and VIII. These findings suggest that alterations in the cerebellar structure in ASD are associated with a reduced ability to integrate online visual feedback information to rapidly update internal action representations that are used to guide initial motor output.

In the present study, we examined the relationships between the volumes of multiple cerebellar subregions and both skeletomotor and oculomotor behaviors in ASD. Regarding skeletomotor control, consistent with our prior behavioral studies (Mosconi et al., 2015a; Wang et al., 2015; Unruh et al., 2021), we predicted that individuals with ASD would show increased force variability. Consistent with the role of cerebellar Crus I in skeletomotor control (Vaillancourt et al., 2006; Proville et al., 2014) and differences between individuals with ASD and TD controls in Crus I function (Unruh et al., 2019; Wang et al., 2019; Lepping et al., 2021), we predicted that force variability increases in ASD would be associated with cerebellar Crus I volumes. Regarding oculomotor control, consistent with previous behavioral studies (Takarae et al., 2004; Stanley-Cary et al., 2011; Schmitt et al., 2014; Unruh et al., 2021), we expected individuals with ASD would show increased saccade error and trial-to-trial error variability. Consistent with previous structural MRI studies (Courchesne et al., 2001; Kaufmann et al., 2003; Stanfield et al., 2008; Webb et al., 2009; Crucitti et al., 2020), we predicted that individuals with ASD would show reduced volume of vermal lobules VI/VII compared to TD controls who would be associated with more severe saccade dysmetria in ASD. Exploratory analyses of separate cerebellar subregions and white matter and their associations with precision gripping variability and saccade error and error variability were also conducted. Based on the findings that cerebellar structural variation may be associated with core ASD symptoms, we also examined the relationships between cerebellar lobular, vermis, and white matter volumes with clinically rated social-communication abnormalities and restricted, repetitive behaviors.

## Materials and Methods

### Participants

Fifty-eight participants with ASD and 34 TD controls matched on age (8–30 years), sex, and handedness completed a structural MRI scan and either precision grip testing, oculomotor testing, or both (Table 1). Due to scheduling constraints and pauses in testing related to COVID-19, not all participants completed all three components of testing. Forty-four participants with ASD and 29 TD controls completed both the MRI scan and precision grip testing. Forty-two participants with ASD and 25 TD controls completed both the MRI scan and oculomotor testing. Participants with ASD were recruited through outpatient clinics. Participants from both groups were recruited through community advertisements and our research registries.

**TABLE 1:** Participant characteristics.

	TD Controls	ASD
N	34 (18 F)	58 (21 F)
Age	17.1 (5.6)	15.7 (4.9)
% right-handed	91.20%	79.30%
Race		
% White	76.5%	82.8%
% Black	5.9%	−
% American Indian or Alaska Native	−	1.7%
% Asian American/Pacific Islander	2.9%	1.7%
% More than one race	11.8%	12.1%
% Not specified or unknown	2.9%	1.7%
Ethnicity		
% Hispanic/Latinx	20.6%	13.8%
Weight in kg	58.2 (20.1)	63.1 (21.8)
Height in cm	161.3 (12.7)	164.5 (13.9)
VIQ	110 (10)	99 (17)
PIQ	114 (10)	100 (17)
Left hand MVC	71.3 (26.8)	57.8 (22.0)
Right hand MVC	70.8 (27.4)	55.0 (20.7)
ADOS CSS	-	5.9 (2.3)
RBS-R Total Score	-	29.5 (19.0)

*ASD, autism spectrum disorder; F, female; VIQ, verbal IQ; PIQ, performance IQ; ADOS CSS, Autism Diagnostic Observation Schedule Calculated Severity Score; RBS-R, Repetitive Behaviors Scale—Revised; Values reported as M (SD).*

All participants with ASD met DSM-5 criteria as determined by the Autism Diagnostic Observation Schedule—Second Edition (ADOS-2; Lord et al., 2012), Autism Diagnostic Interview—Revised (ADI-R; Lord et al., 1994), and expert clinical opinion according to DSM 5 criteria (American Psychiatric Association [APA], 2013). All participants with ASD either had a previous diagnosis of ASD or were strongly suspected to meet the diagnostic criteria for ASD by their medical provider. General cognitive abilities (IQ) were assessed using the Wechsler Abbreviated Scale of Intelligence—Second Edition (WASI-II; Wechsler, 2011). Due to restrictions regarding in-person testing during the COVID-19 pandemic, 20 participants completed a remote administration of the two-subtest version of the WASI-II based on the publisher’s recommendations. Exclusionary criteria for participants with ASD included full-scale IQ less than 60 or a known genetic or metabolic disorder associated with ASD (e.g., fragile X syndrome, tuberous sclerosis complex). Exclusionary criteria for TD controls included the presence of any lifetime psychiatric or neurodevelopmental disorder, or a family history of neurodevelopmental disorders in the first- or second-degree relatives. Exclusionary criteria for both groups included a history of a significant psychiatric disorder (e.g., schizophrenia, bipolar disorder, and personality disorder), meningitis, encephalitis, seizure disorder, or head trauma with loss of consciousness, current use of medications known to affect sensorimotor functioning (e.g., stimulants, benzodiazepines, and anticonvulsants; Reilly et al., 2008), and significant complications during pregnancy, labor, or delivery. All participants refrained from caffeine, nicotine, alcohol, and recreational drug use on the day of testing.

### Precision Grip Testing

**FIGURE 1:**
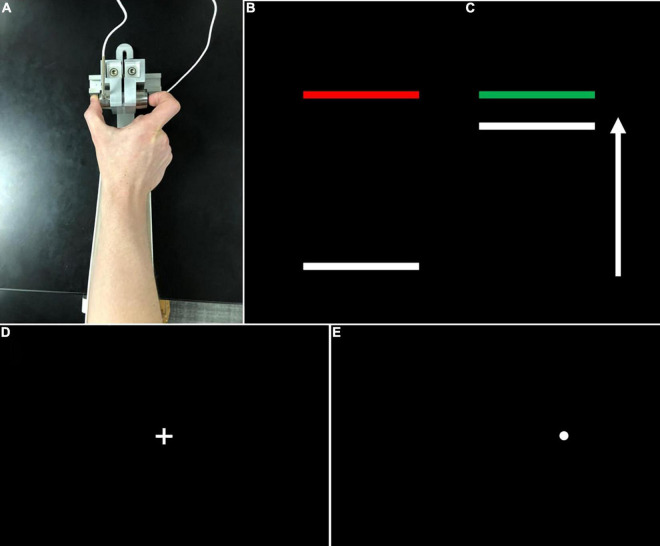
**(A)** Grip configuration and load cells for precision grip testing. Participants pressed with their thumb and forefinger against two precision load cells. Participants pressed the load cells as quickly as possible when the red target bar **(B)** turned green **(C)** and continued pressing to maintain the force bar steady at the level of the green target bar. **(D)** During visually guided saccade testing, participants fixed their gaze on a centrally located crosshair at the start of each trial, then looked quickly toward **(E)** peripheral targets (i.e., white circles) which appeared pseudorandomly at ± 12° or 24° of visual angle.

During precision grip testing, participants were seated 52 cm away from a 69 cm LCD monitor (resolution = 1,366 × 768; refresh rate = 120 Hz). Their hands were pronated and lay flat with digits comfortably extended while gripping two opposing precision ELFF-B4-100N load cells of 1.27 cm in diameter (Measurement Specialties, Hampton, VA, United States) with their thumb and forefinger (Figure 1A). Load cells were secured to custom-made forearm rests mounted to a table (75 cm in height). Electrical resistance changes from the load cells were amplified by four individual resistive bridge strain amplifiers (V72-25; Coulbourn Instruments, Allentown, PA, United States). Amplifier output was sampled continuously at 200 Hz by an analog-to-digital converter (National Instruments, Austin, TX, United States) at 16-bit resolution and converted to Newtons (N) of force using a calibration factor derived from known weights before the study. The system can detect forces down to 0.0016 N.

Before testing, each participant’s maximum voluntary contraction (MVC) was calculated separately for each hand using the average of the maximum force output from three trials in which participants pressed as hard as they could for three seconds (s). MVC trials alternated between hands and were separated by 30 s of rest.

During testing, participants gripped the opposing load cells while viewing two horizontal bars: a horizontal white “force” bar that moved upward with increased force and downward with decreased force and a static target bar that was red during rest (Figure 1B) and turned green to cue the participant to begin pressing at the start of each trial (Figure 1C). Participants received two instructions: (1) to press the load cells as quickly as possible when the red target bar turns green, and (2) to keep pressing so that the force bar stays as steady as possible at the level of the green target bar. The target bar was set at 15% of each participant’s MVC and the visual angle was set at 0.623°. Participants completed the precision grip testing with their left and right hands separately. For each hand, participants completed three 15 s trials separated by 15 s rest periods. The order of hand tested was counterbalanced across participants.

### Oculomotor Testing

Oculomotor testing was administered in a darkened black room using a chinrest positioned 61 cm from a 27-inch BenQ monitor (refresh rate: 144 Hz; resolution: 2,560 × 1,440). Visual stimuli were presented using SR Research Experiment Builder (SR Research Ltd., Ontario, CA, United States), and participants’ eye movements were recorded using an EyeLink 1000 Plus infrared, binocular camera (sampling rate: 500 Hz; accuracy: 0.25–0.5°; SR Research Ltd., Ontario, CA, United States). Participants performed a five-point calibration prior to each block of trials.

Participants completed 60 trials of a visually guided saccade task, separated into two blocks (30 trials per block). During this task, participants fixed their gaze on a centrally located crosshair for 1.5–2 s at the start of each trial (Figure 1D), then were presented peripheral targets (Figure 1E) (i.e., white circles, 0.3° in diameter) at ± 12° or 24° of visual angle for 1.5 ss.

### MRI Data Acquisition

Participants completed a structural MRI scan with a 3T whole-body scanner (Siemens Skyra) and a 32-channel head coil. Participants lay supine with their head stabilized using adjustable padding. A whole-brain T1-weighted (MPRAGE) anatomical scan was acquired across 176 contiguous sagittal slices at 1.200 × 1.055 × 1.055 mm^3^ (FOV 176 × 240 × 256 mm^3^; matrix 176 × 240 × 256 mm^3^; TR = 2.3 s; TE = 2.95 ms; inversion delay to the center k-line 900 ms; flip angle = 9°; pixel bandwidth = 240 Hz; duration 5:12).

### Data Processing

#### Precision Grip Data Processing

Force data were analyzed using custom MATLAB scripts previously developed by our lab (Wang et al., 2015). The force time series was digitally filtered using a fourth-order Butterworth filter and a 15 Hz low-pass cutoff. To assess precision grip performance, the sustained portion of the force timeseries was examined, defined as the 12 s period preceding the appearance of the stop cue (target bar turned from green to red). Portions of the sustained force output in which participants released the force transducers and force output was reduced to zero for greater than 1 s were excluded from analyses. Trials were excluded if they contained less than 8 s of sustained force output following the offset of the initial increase in force, defined as the time-point when the rate of force increase fell below 5% of the peak rate of force increase and the force level was within 90–110% of the mean force of the sustained phase. The peak rate of force increase was defined as the maximum value of the first derivative of the force trace. To assess force variability, the coefficient of variation (CoV) was derived by dividing the standard deviation (SD) of the sustained force time series by the mean of the sustained force time series for each trial.

#### Oculomotor Data Processing

Oculomotor data was filtered prior to scoring using digital finite impulse response filters with non-linear transition bands. Visual inspection of eye movement data was conducted to detect and correct or exclude data confounded by blinks or head movements. The accuracy of the primary saccade was measured as the absolute value of the horizontal distance in degrees of visual angle between the eye location at saccade offset and the target location. The primary saccade was defined as the first saccade that moved at least 20% of the distance to the target. Saccade offset was defined as the timepoint at which the eye velocity fell below 30°/s. Saccades with latencies ≤ 70 ms were considered anticipatory and excluded from analyses. Saccade error variability was calculated as the SD of saccade accuracy across trials.

#### MRI Data Processing

Cerebellar lobules were automatically segmented using the Automatic Cerebellum Anatomical Parcelation using U-Net with Locally Constrained Optimization (ACAPULCO; version 0.2.2) pipeline (Han et al., 2020) and accompanying pediatric template (Carass et al., 2018). Image inhomogeneities were corrected using N4 (Tustison et al., 2010). The 1 mm isotropic ICBM 2009c template was used to register the corrected image to MNI space (Fonov et al., 2011). A bounding box was drawn around the cerebellum, and a modified U-Net was used to segment individual lobules. The parcelated image was transformed back to the original image space and the volume of each region of interest (ROI) was calculated.

**FIGURE 2:**
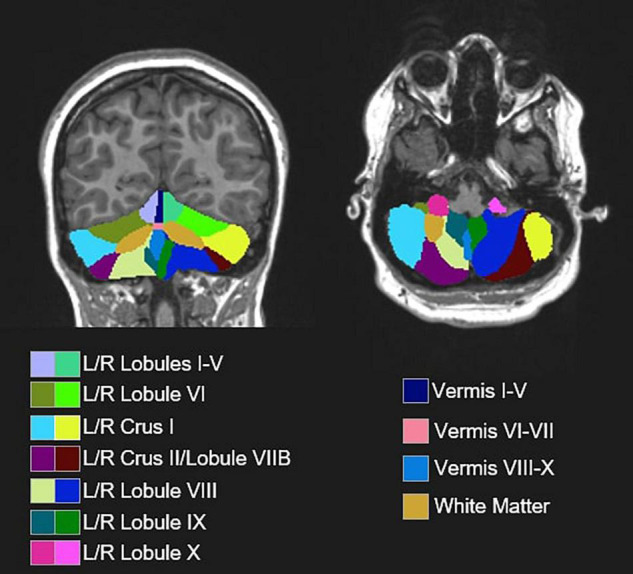
Representative segmentation from a single subject depicting 18 cerebellar ROIs obtained from automated segmentation procedures (ACAPULCO; Han et al., 2020) and an accompanying pediatric template (Carass et al., 2018).

Following automated segmentation, cerebellar parcelations were visually inspected by two separate raters. Discrepancies were discussed and resolved *via* consensus. Extensions of the parcelation into non-cerebellum (e.g., meninges, 4th ventricle) were manually corrected using ITK-SNAP (Yushkevich et al., 2006). Volumes of 18 ROIs were extracted (Figure 2), including separate left and right cerebellar lobules I-V, lobule VI, Crus I, Crus II/lobule VIIB, lobule VIII, lobule IX, and lobule X, as well as vermal lobules I-V, VI-VII, VIII-X, and cerebellar white matter. Raw cerebellar volumes were examined without controlling for total tissue volume since our primary focus was on associations between volumes of individual cerebellar subregions and sensorimotor/clinical behaviors as opposed to the relationships between cerebellar and more diffuse cortical and subcortical structural variations, including regions not thought to be strongly associated with sensorimotor outcomes of interest. Further, given the differences in total tissue volume in ASD (Lange et al., 2015) and distinct scaling factors across the cerebrum and cerebellum (de Jong et al., 2017), normalization to outcomes, such as intracranial volume (ICV) risks incorporating substantial variance unrelated to group differences in the cerebellar volume. Other measures (e.g., body size) possibly affecting the brain size were similar across groups (Table 1).

### Clinical Measures

To assess ASD symptom severity, the calibrated severity score (CSS) from the ADOS-2 was examined. The CSS is an aggregate score ranging from 1 to 10 that allows for the comparison of symptom severity across different ADOS-2 modules. Higher CSS scores reflect more severe ASD symptoms. Caregivers of participants with ASD also completed the repetitive behavior scale-revised (RBS-R; Bodfish et al., 2000; Lam and Aman, 2007), a caregiver-report questionnaire assessing restricted and repetitive behaviors common in ASD. We examined the RBS-R total score. Due to scheduling issues related to the COVID-19 pandemic, not all participants with ASD completed the ADOS-2 and RBS-R. Forty-nine participants with ASD completed the ADOS-2 and 51 completed the RBS-R.

### Statistical Analyses

Linear mixed effects models were used to examine group differences in force variability. Hand (left vs. right) was included as a level one predictor (within-subjects), while group (TD vs. ASD), sex, and age at task administration were included as level two predictors (between-subjects). The group × hand, group × sex, and group × age interaction terms also were examined. Similar linear mixed effects models were used to examine group differences in saccade error and saccade error variability, where target step amplitude (12° vs. 24°) and direction (left vs. right) were included as level one predictors, and group, sex, and age at task administration were included as level two predictors. Associated two- and three-way interaction terms (i.e., group × direction, group × amplitude, group × direction × amplitude) were also examined.

Separate linear mixed effects models were used to examine group differences in the cerebellar volume. For cerebellar hemisphere ROIs (14 ROIs: separate left and right cerebellar lobules I-V, lobule VI, Crus I, Crus II/lobule VIIB, lobule VIII, lobule IX, and lobule X), models included hemisphere (left vs. right) as a level one predictor. Group, sex, and age at MRI administration were included as level two predictors. Associated two- and three-way interaction terms were also examined. Identical models without a hemisphere predictor were used to examine group differences in the cerebellar vermis ROIs and the cerebellar white matter.

Linear mixed effects models were also used to examine group differences in the association between cerebellar volumes and grip and saccade behavior. For force variability, the primary ROIs examined were cerebellar lobules I-V, lobule VI, and Crus I based on prior functional studies documenting the involvement of these subregions in manual motor behavior (Vaillancourt et al., 2006; Stoodley et al., 2012). For absolute error of visually guided saccades, the primary ROI examined was vermal lobules VI-VII based on the known functional role of posterior vermis in the oculomotor control (Ohtsuka and Noda, 1992; Scudder et al., 2002). The Benjamini–Hochberg procedure was used to control the number of models examined at a false-discovery rate of 5% and alpha level of 0.05. We also conducted separate exploratory analyses of associations between behavioral outcomes and the volume of all other cerebellar ROIs. These analyses were considered exploratory and hypothesis-generating, so no Type I error correction was applied. For force variability, behavior-cerebellar volume models included hand as a level one predictor. For saccade error and saccade error variability, amplitude and direction were included as level one predictors. For both sets of models, group, brain volume, and age at MRI administration were included as level two predictors. Sex was included as a covariate of no interest. Three-way interactions (grip: group × hand × volume; oculomotor: group × direction × volume, group × amplitude × volume) and nested two-way interactions were also examined.

For participants with ASD, similar linear mixed effects models were used to examine the associations between cerebellar ROI volumes and ASD severity measured using the ADOS CSS and RBS-R total score. Models included separate hemispheric predictors for the seven homologous ROIs. Separate regression models were used to examine the linear associations between clinical outcomes and the four non-lateralized ROIs. Given distinct patterns of cerebellar development across males and females in TD (Tiemeier et al., 2010) and previously reported sex-specific associations between the cerebellar volume and clinical symptoms (Supekar and Menon, 2015), two-way interaction terms of sex × volume were also examined. These analyses were considered exploratory and hypothesis-generating, so no Type I error correction was applied.

For all analyses, interaction terms were iteratively removed if their inclusion did not improve the model fit, consistent with the best-practice recommendations in maintaining model parsimony (Matuschek et al., 2017). Force variability was log-transformed for all analyses due to its non-normal distribution. All other outcomes were normally distributed. Age was group-mean centered. R version 4.1.0 (“Camp Pontanezen”) was used for all analyses. Mixed effects models were constructed using the *lme4* package (Bates et al., 2015). Simple slopes estimates used to probe interaction effects were obtained using the *interactions* package (Long, 2019).

## Results

### Precision Grip Force

Relative to TD controls, individuals with ASD showed reduced left- [*t*(49.73) = −2.223, *p* = 0.031] and right-hand MVC [*t*(48.57) = −2.639, *p* = 0.011].

Individuals with ASD showed elevated force variability compared to TD controls [*F*_(1,64.75)_ = 17.01, *p* < 0.001]. Increased age was associated with lower force variability [*F*_(1,65.08)_ = 34.040, *p* < 0.001]. Force variability was similar across hands [*F*_(1,67.62)_ = 0.636, *p* = 0.428] and sexes [*F*_(1,64.71)_ = 0.218, *p* = 0.642].

### Saccade Precision

Saccade error was similar for individuals with ASD and TD controls [*F*_(1,59)_ = 0.056, *p* = 0.815]. Increased age was associated with reduced saccade error, though this relationship was at a trend level [*F*_(1,59)_ = 2.819, *p* = 0.098]. Saccade error was the greatest for leftward 24° saccades [amplitude × direction: *F*_(1,192)_ = 21.262, *p* < 0.001]. Saccade error was similar across males and females [*F*_(1,59)_ = 0.234, *p* = 0.631].

Saccade error variability was similar between individuals with ASD and TD controls [*F*_(1,59)_ = 1.739, *p* = 0.192]. Increased age was associated with reduced saccade error variability [*F*_(1,59)_ = 5.393, *p* = 0.024]. Saccade error variability was the greatest for leftward 24° saccades [amplitude × direction: *F*_(1,192)_ = 18.348, *p* < 0.001]. Saccade error variability was similar between males and females [*F*_(1,59)_ = 0.299, *p* = 0.586].

### Cerebellar Volumetrics

#### Cerebellar Hemisphere Lobules

Individuals with ASD and TD controls showed no differences in volumes of cerebellar lobules I-V [Table 2; *F*_(1,87)_ = 1.585, *p* = 0.211]. Males showed greater volumes of cerebellar lobules I-V than females [*F*_(1,87)_ = 17.840, *p* < 0.001]. Volumes of cerebellar lobules I-V were greater for the left compared to the right hemispheres [*F*_(1,90)_ = 4.038, *p* = 0.047], though this difference did not survive correction for multiple comparisons (*p*_*crit*_ = 0.029).

**TABLE 2:** Volume of lateralized regions of interest (ROIs).

	Lobules I-V	Lobule VI	Crus I	Crus II/Lobule VIIB	Lobule VIII	Lobule IX	Lobule X
Controls							
*Left*	6.57 (0.93)	10.85 (1.44)	15.53 (1.96)	15.45 (2.18)	12.32 (1.74)	3.72 (0.67)	0.61 (0.11)
*Right*	6.47 (0.98)	10.45 (1.48)	15.95 (2.04)	14.35 (2.07)	12.70 (1.63)	3.64 (0.67)	0.60 (0.09)
ASD							
*Left*	6.48 (0.99)	10.82 (1.18)	15.85 (2.05)	15.34 (2.03)	12.38 (1.51)	3.71 (0.66)	0.59 (0.10)
*Right*	6.33 (0.93)	10.68 (1.20)	15.92 (2.34)	14.02 (2.34)	13.21 (1.94)	3.68 (0.66)	0.62 (0.09)

*Values reported as M (SD); Volume is reported as cm^3^.*

Individuals with ASD and TD controls showed no differences in volumes of cerebellar lobule VI [*F*_(1,86)_ = 0.003, *p* = 0.958]. Group differences in the volume of cerebellar lobule VI varied across sexes [group × sex: *F*_(1,86)_ = 5.610, *p* = 0.020], but this finding did not survive the correction for multiple comparisons (*p*_*crit*_ = 0.014). Specifically, males with ASD showed reduced volumes relative to TD males [*t*(86) = −1.686, *p* = 0.337], while females with ASD showed greater volumes relative to TD females [*t*(86) = 1.676, *p* = 0.343]. Volumes of lobule VI were greater for the left compared to the right hemisphere [*F*_(1,90)_ = 4.953, *p* = 0.029], but this difference did not survive the correction for multiple comparisons (*p*_*crit*_ = 0.021).

Individuals with ASD and TD controls showed no differences in volumes of cerebellar Crus I [*F*_(1,87)_ = 0.117, *p* = 0.733]. Males showed greater volumes of cerebellar Crus I than females [*F*_(1,87)_ = 15.276, *p* < 0.001].

**FIGURE 3:**
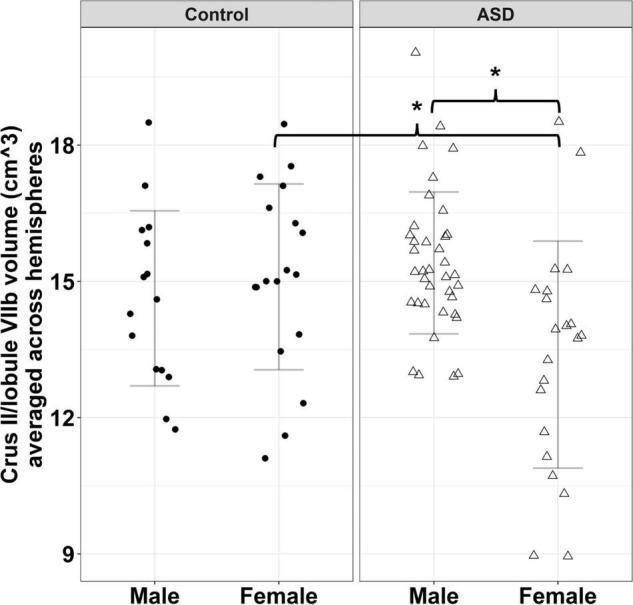
Sex-dependent group differences in Crus II/lobule VIIB volume. Error bars reflect mean ± 1 SD. * denotes *p* < 0.05.

Group differences in the volume of cerebellar Crus II/lobule VIIB varied across sexes [Figure 3; group × sex: *F*_(1,86)_ = 7.570, *p* = 0.007]. Specifically, females with ASD showed reduced volumes of Crus II/lobule VIIB relative to TD females [*t*(86) = −2.625, *p* = 0.049], while males with ASD and TD showed similar volumes [*t*(86) = 1.245, *p* = 0.600]. Volumes of Crus II/lobule VIIB were greater for the left compared to the right hemisphere [*F*_(1,90)_ = 84.119, *p* < 0.001].

Males showed greater volumes of lobule VIII than females [*F*_(1,87)_ = 45.560, *p* < 0.001]. Individuals with ASD showed differences in the volumes of lobule VIII that varied as a function of hemisphere [group × hemisphere: *F*_(1,89)_ = 4.890, *p* = 0.030], though this finding did not survive the correction for multiple comparisons (*p*_*crit*_ = 0.007). Specifically, relative to TD controls, individuals with ASD showed smaller volumes of left lobule VIII [*t*(110) = −0.858, *p* = 0.827], but greater volumes of right lobule VIII [*t*(110) = 0.657, *p* = 0.913].

Individuals with ASD and TD controls showed no differences in volumes of cerebellar lobule IX [*F*_(1,87)_ = 0.124, *p* = 0.726]. Males showed greater volumes of lobule IX than females [*F*_(1,87)_ = 16.841, *p* < 0.001].

Individuals with ASD and TD controls showed no differences in volumes of cerebellar lobule X [*F*_(1,87)_ = 0.068, *p* = 0.795]. Males showed greater volumes of lobule X than females [*F*_(1,87)_ = 20.908, *p* < 0.001].

#### Cerebellar Vermis and White Matter

Individuals with ASD showed reduced volumes of cerebellar vermal lobules I-V relative to TD controls, though this effect was at a trend level [*F*_(1,87)_ = 3.718, *p* = 0.057]. Males showed greater volumes of vermal lobules I-V than females [*F*_(1,87)_ = 19.252, *p* < 0.001].

**FIGURE 4:**
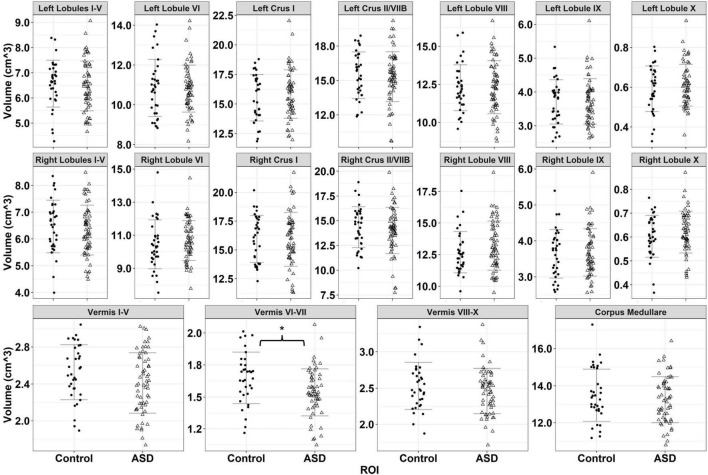
Volumes for 18 cerebellar ROIs for typically developing controls (black circles) and individuals with ASD (empty triangles). Error bars reflect mean ± 1 SD. * denotes *p* < 0.05.

Relative to TD controls, individuals with ASD showed reduced volumes of cerebellar vermal lobules VI-VII [Figure 4 and Table 3; *F*_(1,87)_ = 8.119, *p* = 0.005]. Males showed greater volumes of vermal lobules VI-VII than females [*F*_(1,87)_ = 4.708, *p* = 0.033].

**TABLE 3:** Volume of vermal and white matter ROIs.

	Vermal lobules I-V	Vermal lobules VI-VII	Vermal lobules VIII-X	White matter
Controls	2.53 (0.30)	1.65 (0.20)	2.53 (0.32)	13.49 (1.41)
ASD	2.41 (0.33)	1.54 (0.18)	2.46 (0.31)	13.25 (1.25)

*Values reported as M (SD); Volume is reported as cm^3^.*

Individuals with ASD and TD controls showed similar volumes of vermal lobules VIII-X [*F*_(1,87)_ = 0.960, *p* = 0.330]. Males showed greater volumes of vermal lobules VIII-X than females [*F*_(1,87)_ = 8.118, *p* = 0.005].

Individuals with ASD and TD controls showed similar volumes of cerebellar white matter [*F*_(1,86)_ = 0.892, *p* = 0.348]. Males showed greater volumes of cerebellar white matter than females [*F*_(1,86)_ = 24.919, *p* < 0.001]. Increased age was associated with greater volume of cerebellar white matter for TD controls (β = 0.129, *p* < 0.001), but not individuals with ASD (β = 0.035, *p* = 0.268), though this finding did not survive the correction for multiple comparisons [group × age: *F*_(1,86)_ = 3.971, *p* = 0.049; *p*_*crit*_ = 0.013].

### Cerebellar Volume and Sensorimotor Behavior

#### Cerebellar Associations With Precision Grip Force Variability

Force variability was not associated with volumes of right lobules I-V [*F*_(1,66.57)_ = 0.461, *p* = 0.500], right lobule VI [*F*_(1,66.62)_ = 0.013, *p* = 0.910], left lobules I-V [*F*_(1,66.50)_ = 0.431, *p* = 0.514], or left lobule VI [*F*_(1,66.52)_ = 0.373, *p* = 0.544]. Increased force variability was associated with increased volume of both right Crus I [*F*_(1,68.73)_ = 7.737, *p* = 0.007] and left Crus I [*F*_(1,69.78)_ = 8.312, *p* = 0.005].

Exploratory analyses of associations between non-skeletomotor cerebellar ROIs and precision grip behavior indicated that the relationship between the volume of right lobule VIII and force variability varied across hands [hand × volume: *F*_(1,67.27)_ = 5.278, *p* = 0.025], reflecting the finding that increased volume of right lobule VIII was associated with reduced left- (β = −0.097, *p* = 0.026) but not right-hand force variability (β = −0.035, *p* = 0.409). Similarly, the relationship between volume of left lobule VIII and force variability varied across hands hand × volume: *F*_(1,67.14)_ = 4.943, *p* = 0.030], such that increased volume of left lobule VIII was associated with reduced left- (β = −0.074, *p* = 0.121) but not right-hand force variability (β = −0.007, *p* = 0.876). Increased right lobule IX [*F*_(1,69.86)_ = 2.875, *p* = 0.094] and left lobule IX [*F*_(1,70.39)_ = 3.221, *p* = 0.077] volumes were associated with increased force variability, though these relationships were at a trend level. Increased cerebellar white matter volume was associated with reduced force variability [*F*_(1,66.67)_ = 6.212, *p* = 0.015]. No other associations between cerebellar volume and force variability were significant, and all associations between force variability and cerebellar volume were similar across groups.

#### Cerebellar Associations With Saccade Error

**FIGURE 5:**
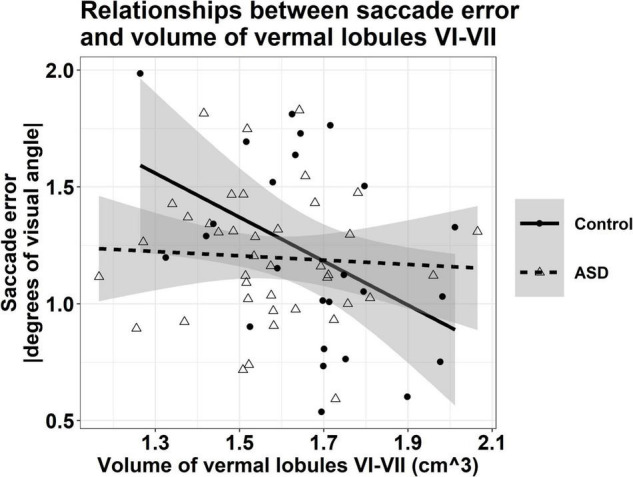
Relationship between the volume of vermal lobules VI-VII and saccade error, averaged across target direction and amplitude. Greater volumes of vermal lobules VI-VII were associated with reduced saccade error in TD controls (*r* = –0.438, *p* = 0.007), but not individuals with ASD (*r* = –0.060, *p* = 0.671). Shaded regions reflect the 95% confidence interval of a group-level linear fit.

Associations between the volumes of vermal lobules VI-VII and saccade error varied across groups, though this interaction was at a trend level [Figure 5; group × volume: *F*_(1,60)_ = 3.896, *p* = 0.053]; greater volumes of vermal lobules VI-VII were associated with reduced saccade error in TD controls (β = −1.022, *p* = 0.007), but not individuals with ASD (β = −0.124, *p* = 0.671).

Exploratory analyses indicated that the relationships between saccade error and volume of right Crus II/lobule VIIB varied as a function of group and target step amplitude [group × amplitude × volume: *F*_(1,189)_ = 6.781, *p* = 0.010]. At 24° only, increased volume of right Crus II/lobule VIIB was associated with lower saccade error for TD controls (β = −0.129, *p* < 0.001), while greater volume was associated with more severe saccade error for individuals with ASD (β = 0.065, *p* = 0.021). The relationships between saccade error and volume of left Crus II/lobule VIIB also varied as a function of group and target step amplitude [group × amplitude × volume: *F*_(1,189)_ = 9.662, *p* = 0.002]. At 24° only, increased left Crus II/lobule VIIB volume was associated with lower saccade error for TD controls (β = −0.117, *p* = 0.002), while increased volume was associated with greater error for individuals with ASD (β = 0.092, *p* = 0.003).

Increased volume of right lobule VIII was associated with lower saccade error for TD controls (β = −0.107, *p* = 0.008) but not for individuals with ASD [β = −0.016, *p* = 0.590; group × volume: *F*_(1,60)_ = 4.151, *p* = 0.046]. The relationships between saccade error and volume of left lobule VIII varied as a function of group and target step amplitude [group × amplitude × volume: *F*_(1,189)_ = 9.213, *p* = 0.003]. Increased volume of left lobule VIII was associated with lower saccade error for TD controls across 12° (β = −0.113, *p* = 0.018) and 24° (β = −0.239, *p* < 0.001), while increased volume was only related to saccade error for individuals with ASD at 12° (β = −0.077, *p* = 0.038) but not 24° (β = 0.011, *p* = 0.758).

The relationships between saccade error and volume of right lobule X also varied as a function of group and target step amplitude [group × amplitude × volume: *F*_(1,189)_ = 6.285, *p* = 0.013]. At 24° only, the increased volume of right lobule X was associated with lower saccade error for TD controls (β = −3.178, *p* = 0.016), while increased volume was not related to saccade error for individuals with ASD (β = −0.095, *p* = 0.912).

The relationships between saccade error and volume of vermal lobules I-V varied as a function of group and target step amplitude [group × amplitude × volume: *F*_(1,189)_ = 6.298, *p* = 0.013]. At 24° only, increased volume of vermal lobules I-V was associated with lower saccade error for TD controls (β = −0.641, *p* = 0.026), while increased volume was not related to saccade error for individuals with ASD (β = 0.252, *p* = 0.255).

Increased cerebellar white matter volume was associated with lower saccade error, though this relationship was at a trend level [*F*_(1,61)_ = 2.799, *p* = 0.099].

#### Cerebellar Associations With Saccade Error Variability

The relationships between saccade error variability and volumes of vermal lobules VI-VII varied as a function of group and target direction [group × direction × volume: *F*_(1,189)_ = 4.111, *p* = 0.044]. Greater volumes of vermal lobules VI-VII were associated with reduced *leftward* saccade error variability for TD controls (β = −0.137, *p* = 0.609) and individuals with ASD (β = −0.185, *p* = 0.385), while greater volume was associated with lower *rightward* saccade error variability for TD controls (β = −0.416, *p* = 0.123) but not individuals with ASD (β = 0.256, *p* = 0.231).

Exploratory analyses indicated that increased volume of right Crus II/lobule VIIB was associated with lower saccade error variability for TD controls (β = −0.043, *p* = 0.017) but not for individuals with ASD [β = 0.005, *p* = 0.722; group × volume: *F*_(1,60)_ = 4.488, *p* = 0.038].

Increased right [*F*_(1,61)_ = 5.647, *p* = 0.021] and left lobule VIII volumes [*F*_(1,61)_ = 15.050, *p* < 0.001] were associated with lower saccade error variability.

The relationships between saccade error variability and volume of right lobule X varied as a function of group and target step amplitude [group × amplitude × volume: *F*_(1,187)_ = 4.214, *p* = 0.041]. For TD controls, increased volume of right lobule X was associated with greater saccade error variability at 12° (β = 0.665, *p* = 0.380) but lower saccade error variability at 24° (β = −0.519, *p* = 0.493). For individuals with ASD, increased volume of right lobule X was associated with lower saccade error variability at 12° (β = −0.522, *p* = 0.294) but greater saccade error variability at 24° (β = 0.208, *p* = 0.675).

Increased volume of cerebellar white matter was associated with lower saccade error variability, though this relationship was at a trend level [*F*_(1,61)_ = 3.965, *p* = 0.051].

### Cerebellar Associations With Autism Spectrum Disorder Severity

**FIGURE 6:**
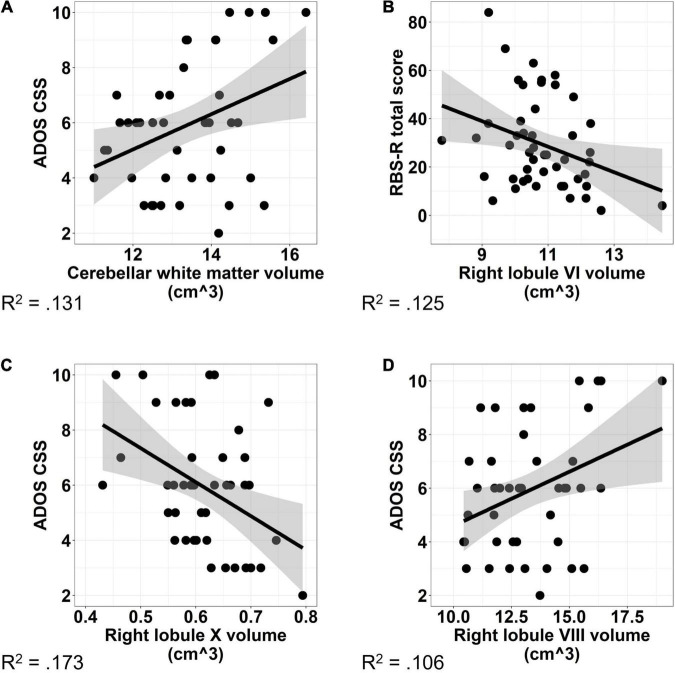
Associations between cerebellar volume and ASD symptom severity. Increased volume of cerebellar white matter **(A)** and right lobule VIII **(B)** were associated with increased ASD symptom severity as measured using the ADOS-2 Calibrated Severity Score. Increased volume of right lobule X **(C)** was associated with reduced ASD symptom severity. Increased volume of right lobule VI **(D)** was associated with reduced severity of RRBs as measured using the Repetitive Behaviors Scale—Revised. The shaded region reflects the 95% confidence interval of a linear fit. R^2^ values reflect the proportion of variance in clinical symptoms accounted for by the cerebellar volume in a one-term model. ADOS CSS, Autism Diagnostic Observation Schedule Calibrated Severity Score; RBS-R, Repetitive Behaviors Scale—Revised.

**FIGURE 7:**
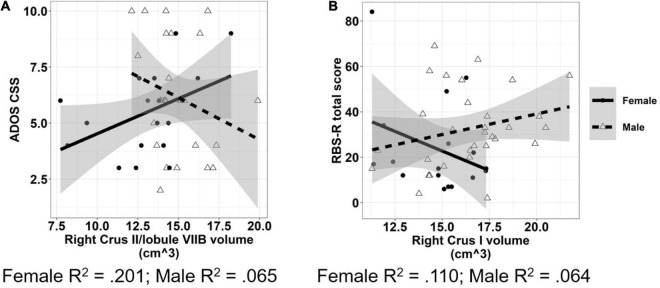
Associations between cerebellar volumes and ASD symptom severity which vary across males and females. Increased volume of cerebellar right Crus II/lobule VIIB **(A)** was associated with reduced ASD symptom severity in males, but not females. Increased volume of cerebellar right Crus I **(B)** was associated with reduced severity of RRBs in females, but not males. The shaded region reflects the 95% confidence interval of a linear fit. R^2^ values reflect the proportion of variance in clinical symptoms accounted for by cerebellar ROI volume in a one-term model. ADOS CSS, Autism Diagnostic Observation Schedule Calibrated Severity Score; RBS-R, Repetitive Behaviors Scale—Revised.

Increased volumes of cerebellar white matter [Figure 6A; *F*_(1,42)_ = 6.331, *p* = 0.016] and right lobule VIII [Figure 6B; *F*_(1,41)_ = 6.044, *p* = 0.018] were associated with more severe clinically rated ASD symptoms (ADOS-CSS). Smaller volumes of right lobule X were associated with more severe ASD symptoms [Figure 6C; *F*_(1,41)_ = 9.887, *p* = 0.003]. The relationship between right Crus II/lobule VIIB volume and ASD symptom severity varied across males and females with ASD; smaller volumes were associated with more severe ASD symptoms in males (β = −0.688, *p* = 0.033), but not females [Figure 7A; β = −0.023, *p* = 0.935; sex × volume: *F*_(1,40)_ = 4.381, *p* = 0.043].

Increased volume of right lobule VI was associated with reduced clinically rated RRB severity as measured by the RBS-R [Figure 6D; *F*_(1,44)_ = 5.484, *p* = 0.024]. The relationship between volume of right Crus I and severity of RRBs varied across males and females with ASD; increased volume of right Crus I was associated with reduced severity of RRBs in females (β = −6.488, *p* = 0.048), but not males [Figure 7B; β = −1.077, *p* = 0.675; sex × volume: *F*_(1,43)_ = 4.210, *p* = 0.046].

## Discussion

We found that associations between cerebellar structure and sensorimotor behaviors vary across effector systems and are different in ASD and TD controls, suggesting atypical cerebellar development is associated with multiple sensorimotor difficulties in ASD. We also document differences in cerebellar volumes in individuals with ASD relative to TD controls which varied across subregions and between males and females. Specifically, we found that cerebellar volumetric reductions in ASD compared to TD controls were specific to vermal lobules VI-VII, consistent with prior studies (Stanfield et al., 2008; Crucitti et al., 2020). We also found that females with ASD showed reduced volumes of bilateral cerebellar Crus II/lobule VIIB relative to TD females, while TD males and males with ASD showed similar volumes, indicating females with ASD may show distinct patterns of neuropathology relative to males with ASD. We also show that volumes of cerebellar white matter and right lobules VI, VIII, and X each are associated with clinically rated ASD symptoms, suggesting that cerebellar structural differences may play a role in core features of the disorder(s). Last, we show that reduced volume of right Crus I was associated with more severe restricted and repetitive behaviors females only, while reduced volume of right Crus II was associated with greater ASD symptom severity in males only, suggesting that cerebellar correlates of clinical symptoms may be sex-specific.

### Volumetrics of Discrete Cerebellar Subregions in Autism Spectrum Disorder

We found that individuals with ASD show reduced volume of vermal lobules VI-VII relative to TD controls, consistent with multiple prior studies and meta-analyses (Townsend et al., 1999; Kaufmann et al., 2003; Stanfield et al., 2008; Stoodley, 2014; Crucitti et al., 2020). Vermal lobules VI-VII (“oculomotor vermis”) Purkinje cells innervate caudal fastigial nuclei cerebellar output to brainstem movement cells that initiate eye movements (Ohtsuka and Noda, 1992; Scudder et al., 2002). Ablation of oculomotor vermis in non-human primates increases saccade error and saccade error variability independent of damage to cerebellar nuclei, highlighting the role of oculomotor vermis in encoding amplitude information to maximize saccade accuracy (Takagi et al., 1998). Our findings converge with previous reports of reduced activation of oculomotor vermis and cerebellar hemispheres during visually guided saccades in ASD relative to TD controls to implicate abnormal structural development of oculomotor vermis in ASD (Takarae et al., 2007). Histopathological findings suggest that vermal hypoplasia in ASD may reflect perinatal loss of Purkinje cells postmigration (Whitney et al., 2009) or postnatal disruptions in granular cell migration (Courchesne et al., 1988) suggesting neurodevelopmental processes contributing to cerebellar pathology and risk of ASD begins early in ontogeny.

We also found that females with ASD show reduced volume of bilateral cerebellar Crus II/lobule VIIB relative to males with ASD and TD females. Reduced volume of cerebellar Crus II has been reported in school-aged children (8–13 years) with ASD across sexes (D’Mello et al., 2016), though studies of younger children (2–7 years) have indicated right Crus II volumes are selectively *increased* in females with ASD (Retico et al., 2016). While we did not examine group by sex by age interactions for structural outcomes due to the relatively small number of females with ASD at younger ages in our sample, these findings together implicate the early overgrowth of Crus II in female patients may be followed by a period of attenuated growth relative to TD during middle childhood and into adulthood. This neurodevelopmental pattern would be consistent with trajectories of total brain volumes in ASD that appear to be characterized by early overgrowth in the first years of life followed by slowed growth and reduced volumes in adulthood (Courchesne et al., 2011). Given the dense connectivity between Crus II and prefrontal cortex (PFC) *via* dentate nuclei and thalamus (O’Reilly et al., 2010; Stoodley et al., 2012), these results are also consistent with a prior study showing that early cortical overgrowth in ASD may be more severe in PFC networks (Carper et al., 2002).

### Associations Between Cerebellar Structure and Sensorimotor Behaviors in Autism Spectrum Disorder

We replicate our prior findings that individuals with ASD show increased force variability during precision gripping (Mosconi et al., 2015a; Wang et al., 2015) and extend these results by demonstrating that increases in force variability are associated with increased bilateral Crus I and reduced cerebellar white matter volumes. Along with lobules V/VI, cerebellar Crus I shows selective involvement in the control of hand movements and increased activation during precision gripping (Vaillancourt et al., 2006; Neely et al., 2013). These prior results highlight functional gradients that cut across anatomically defined cerebellar subregions (Guell et al., 2018) but, combined with our results, these indicate structural variations associated with atypical skeletomotor behavior in ASD are relatively circumscribed to posterior-lateral cerebellum. Innervation of Crus I from sensory cortices *via* pontine nuclei supports reactive adjustments of motor output translated to motor cortex through thalamus (Proville et al., 2014). Alterations of Crus I anatomy may disrupt the integration of sensory feedback during sustained motor actions as during our test of continuous precision grip force. Consistent with this hypothesis, we have found abnormal Crus I functional connectivity with visuomotor cortical targets, including posterior parietal and frontal/prefrontal cortices, associated with increased force variability in ASD during rest (Wang et al., 2019) and precision gripping (Lepping et al., 2021). Altered Crus I functional connectivity with medial PFC and inferior parietal cortex also appears to be strongly associated with separate clinical dimensions of ASD including both social-communication challenges and increased severity of repetitive behaviors as demonstrated by both patient and mouse genetic model studies (Stoodley et al., 2017; Tsai et al., 2018; Kelly et al., 2020). These results suggest a critical role of Crus I in the development of multiple clinical traits associated with ASD and implicate posterior-lateral cerebellar networks as strong candidates for targeted therapeutics aimed at improving sensorimotor and associated developmental outcomes.

Consistent with previously reported associations between increased cerebellar white matter and greater finger tapping speed and manual dexterity in TD (Koppelmans et al., 2015), our study indicates that white matter volume is associated with precision motor performance in ASD. Diffusion tensor imaging (DTI) studies of individuals with ASD have shown reduced microstructural integrity of cerebellar white matter, including increased mean diffusivity of middle cerebellar peduncles, the primary cortical-brainstem afferent pathway to cerebellum (Groen et al., 2011), and reduced fractional anisotropy and increased mean diffusivity of superior cerebellar peduncles, the primary efferent pathway to cerebral cortex from cerebellum (Catani et al., 2008; Sivaswamy et al., 2010). Our findings add to this literature by demonstrating that dysmaturation of cerebellar white matter is associated with reduced fine motor precision and suggest that intracerebellar structural connectivity alterations in ASD may contribute to difficulties with sensorimotor control.

Our finding that greater volumes of vermal lobules VI-VII were associated with reduced saccade error and rightward saccade error variability in TD controls is consistent with prior human and non-human primate studies demonstrating the selective involvement of posterior vermis in modulating the precision of saccadic eye movements (Vahedi et al., 1995; Takagi et al., 1998; Golla et al., 2008). In contrast, volumes of vermal lobules VI-VII were not associated with saccade accuracy in ASD, suggesting that cerebellar correlates of saccade amplitude precision are different in ASD. The limited association of volumes of vermal lobules VI-VII and oculomotor control in ASD may reflect increased involvement of separate cortical or subcortical systems in supporting eye movement precision in patients as suggested by a prior functional MRI study of saccades (Takarae et al., 2007). Specifically, studying visually guided saccades in adults with ASD, Takarae et al. (2007) showed reduced activation of cerebellar vermis and hemispheres in ASD relative to TD controls, but increased activation of the dorsolateral PFC, caudate, thalamus, and the anterior cingulate cortex in patients suggesting frontostriatal networks may compensate for atypical function in cerebellar motor systems in ASD. Combined with our findings of reduced volume of vermal lobules VI-VII in ASD and similar oculomotor performance across ASD and TD, as well as evidence that alterations in posterior vermis likely emerge early in neurodevelopment in ASD (Courchesne et al., 1988; Whitney et al., 2009; Crucitti et al., 2020), these prior functional MRI findings combine with our structural MRI-sensorimotor results to suggest reorganization of cortical and subcortical systems in ASD may compensate for early emerging pathology of the oculomotor vermis in ASD to support the control of saccadic eye movements. It is also possible that the differential associations between volumes of oculomotor vermis and saccade precision in ASD and TD reflect unique features of our ASD sample. Specifically, we did not see oculomotor differences in our ASD sample, in contrast to multiple prior studies from our group and others (Takarae et al., 2004; Luna et al., 2007; Johnson et al., 2012; Schmitt et al., 2014; Unruh et al., 2021). These differences may reflect a limited range of cognitive abilities in our sample that only included individuals who could complete both eye movement and MRI procedures. Differences in saccade accuracy may also vary across other demographic or clinical characteristics; for example, we studied a greater proportion of females with ASD relative to previous studies (Johnson et al., 2012; Unruh et al., 2021).

### Associations With Clinical Symptoms

Our findings of associations between clinically rated ASD symptom severity and volumes of multiple cerebellar subregions, including right cerebellar lobules VI, VIII, and X, as well as cerebellar white matter are consistent with the known role of cerebellum in the modulation of cognitive and social-communicative functions (Schmahmann et al., 2007). Through reciprocal cerebellar-cortical circuits, the cerebellum serves to modify skilled sensorimotor actions (Stein and Glickstein, 1992), emotional expression (Aarsen et al., 2004), and social behaviors (Kelly et al., 2020) affected in ASD. Differences across these areas may reflect difficulties integrating cortical feedback with internal models and prior expectations to refine output. As multiple networks (e.g., cognitive control, default mode network, and sensorimotor) are represented within individual cerebellar lobules (Buckner et al., 2011; Guell et al., 2018), even circumscribed cerebellar pathology may have downstream effects on the developing brain by impacting multiple cortical networks and developmental abilities (for review, see Stoodley and Limperopoulos, 2016). Our findings highlight a key relationship between cerebellar structural integrity and neurodevelopmental outcomes while adding to a growing body of literature documenting associations between cerebellar anatomy and core features of ASD (Rojas et al., 2006; Riva et al., 2013; D’Mello et al., 2015). Further, and consistent with previous studies, we also demonstrate that associations between cerebellar structure and core ASD symptoms may vary across males and females (Supekar and Menon, 2015), implicating sex-specific clinical correlates of cerebellar pathology.

### Limitations and Future Directions

Our study has several limitations that should be addressed in future studies. First, inclusion of a greater number of females will be important given sex-specific patterns of cerebellar development in TD (Tiemeier et al., 2010) and our findings that sensorimotor and clinical associations vary as a function of sex in ASD. Second, while we examined the functional correlates of cerebellar structural variation across a relatively wide age range, longitudinal studies are needed to clarify the patterns of cerebellar subregions over development and their associations with sensorimotor and clinical outcomes. Previous research suggests cerebellar volumetric differences and their association with clinical symptoms vary across development in ASD (D’Mello et al., 2016; Retico et al., 2016). For example, while vermal hypoplasia is present early in development and persists into adolescence in ASD, associations with clinical symptoms appear to be more readily detectable in older children (Webb et al., 2009; Riva et al., 2013; D’Mello et al., 2015). While we did not examine age-associated variations in brain-behavior associations due to our limited power to detect higher order age-associated interactions, the mean age of participants in our sample was in adolescence and therefore may have amplified associations between the structural variation and clinical symptoms.

### Conclusion

Studying relationships between multiple cerebellar subregions and both skeletomotor and oculomotor behavior in individuals with ASD, we found that increased force variability is associated with increased volume of bilateral Crus I, whereas reduced saccade error is associated with increased volume of vermal lobules VI/VII in TD controls but not individuals with ASD. These findings suggest associations between sensorimotor behavior and cerebellar structure vary across subregions and effector systems in ASD. We also document associations between core clinical symptoms of ASD and volumes of multiple cerebellar subregions, several of which are sex-specific, suggesting that cerebellar pathology may have wide ranging impacts on development in ASD that need to be understood in the context of significant heterogeneity across the autism spectrum.

## Data Availability Statement

The raw behavioral data supporting the conclusions of this manuscript will be made available by the authors, without undue reservation, to any qualified researcher. Structural MRI and clinical data are publicly available through the National Database for Autism Research (NDAR; https://nda.nih.gov/edit_collection.html?id=2711).

## Ethics Statement

This study was carried out in accordance with the recommendations of the University of Kansas Medical Center Institutional Review Board with written informed consent from all subjects. Caregivers of minor participants gave written informed consent, minor participants gave written assent, and adult participants gave written informed consent in accordance with the Declaration of Helsinki.

## Author Contributions

MM was responsible for the conception and design of the study. WM and SK performed the clinical evaluations under the supervision of MM. WM and SK collected the behavioral and MRI data and scored the raw data. WM, SK, and MM performed the statistical analyses. WM, SK, KU, RS, JS, MS, and MM drafted and edited the manuscript. All authors interpreted the results and approved the final version of the manuscript.

## Conflict of Interest

The authors declare that the research was conducted in the absence of any commercial or financial relationships that could be construed as a potential conflict of interest.

## Publisher’s Note

All claims expressed in this article are solely those of the authors and do not necessarily represent those of their affiliated organizations, or those of the publisher, the editors and the reviewers. Any product that may be evaluated in this article, or claim that may be made by its manufacturer, is not guaranteed or endorsed by the publisher.
