# Corrigendum: Cerebellar volumes and sensorimotor behavior in autism spectrum disorder

**DOI:** 10.3389/fnint.2022.1020980

**Published:** 2022-09-09

**Authors:** Walker S. McKinney, Shannon E. Kelly, Kathryn E. Unruh, Robin L. Shafer, John A. Sweeney, Martin Styner, Matthew W. Mosconi

**Affiliations:** ^1^Schiefelbusch Institute for Life Span Studies and Kansas Center for Autism Research and Training (K-CART), University of Kansas, Lawrence, KS, United States; ^2^Clinical Child Psychology Program, University of Kansas, Lawrence, KS, United States; ^3^Department of Psychology, University of Kansas, Lawrence, KS, United States; ^4^Department of Psychiatry and Behavioral Neuroscience, University of Cincinnati College of Medicine, Cincinnati, OH, United States; ^5^Department of Psychiatry and Computer Science, University of North Carolina at Chapel Hill, Chapel Hill, NC, United States

**Keywords:** cerebellum, volumetry, autism spectrum disorder (ASD), sensorimotor, oculomotor, MRI, structure, Crus I

In the published article, there was an error. The MRI sequence parameters provided in the original text were incorrect. A correction has been made to **Materials and Methods**, “*MRI Data Acquisition*,” Paragraph 1:

“Participants completed a structural MRI scan with a 3T whole-body scanner (Siemens Skyra) and a 32-channel head coil. Participants lay supine with their head stabilized using adjustable padding. A whole-brain T1-weighted (MPRAGE) anatomical scan was acquired across 176 contiguous sagittal slices at 1.200 × 1.055 × 1.055 mm^3^ (FOV 176 × 240 × 256 mm^3^; matrix 176 × 240 × 256 mm^3^; TR = 2.3 s; TE = 2.95 ms; inversion delay to the center k-line 900 ms; flip angle= 9°; pixel bandwidth = 240 Hz; duration 5:12).”

The authors apologize for this error and state that this does not change the scientific conclusions of the article in any way. The original article has been updated.

## Publisher's note

All claims expressed in this article are solely those of the authors and do not necessarily represent those of their affiliated organizations, or those of the publisher, the editors and the reviewers. Any product that may be evaluated in this article, or claim that may be made by its manufacturer, is not guaranteed or endorsed by the publisher.

